# Rheological Properties and Thermal Conductivity of Epoxy Resins Filled with a Mixture of Alumina and Boron Nitride

**DOI:** 10.3390/polym11040597

**Published:** 2019-04-02

**Authors:** Van-Dung Mai, Dae-Il Lee, Jun-Hong Park, Dai-Soo Lee

**Affiliations:** 1 Division of Semiconductor and Chemical Engineering, Chonbuk National University, Baekjedaero 567, Deokjin-gu, Jeonju, Chonbuk 54896, Korea; dungmv1983@gmail.com (V.-D.M.); ldi4084@naver.com (D.-I.L.); 2 R & D Center, Lotte Advanced Materials, Sandan-ro 334-27, Yeosu 59616, Korea

**Keywords:** alumina, boron nitride, epoxy polymer composite, rheological properties, thermal conductivity, alumina/boron nitride hybrid

## Abstract

Electronic packaging materials with high thermal conductivity and suitable viscosity are necessary in the manufacturing of highly integrated electronic devices for efficient heat dissipation during operation. This study looked at the effect of boron nitride (BN) platelets on the rheology and thermal conductivity of composites based on alumina (Al_2_O_3_) and epoxy resin (EP) for the potential application as electronic packaging. The viscosity and thermal conductivity of the composite were increased upon increasing filler content. Furthermore, thermal conductivity of the BN/Al_2_O_3_/EP was much higher than that of Al_2_O_3_/EP at almost the same filler loadings. These unique properties resulted from the high thermal conductivity of the BN and the synergistic effect of the spherical and plate shapes of these two fillers. The orientation of BN platelets can be controlled by adjusting their loading to facilitate the formation of higher thermally conductive pathways. The optimal content of the BN in the Al_2_O_3_/EP composites was confirmed to be 5.3 vol %, along with the maximum thermal conductivity of 4.4 W/(m·K).

## 1. Introduction

In electronic systems, packaging materials serve as a key component to protect the device with improved mechanical properties and controlled structural profiles, thereby offering thermal conductive and dissipative pathways, as well as guarding the electronic circuits against environment effects, including humidity, heat, chemicals, and radiation. Therefore, the development of electronic packaging materials with good mechanical properties, high thermal conductivity, high electrical resistance, and a low coefficient of thermal expansion is necessary to produce efficient electronic devices. Currently, epoxy (EP) resins are widely used for packaging of electronic devices because of their excellent electrical insulation properties and heat resistance [1,2,3,4]. However, because of its low thermal conductivity and high thermal expansion, the applications of EP resin are limited. In order to improve the thermal conductivity and lower the thermal expansion of the EP resin, various solutions were studied recently. In this context, the addition of an inorganic filler into an EP matrix to form effective composites is a potential route to improve the performance of the final product. For this purpose, many types of inorganic or metallic fillers with high thermal conductivity, low thermal expansion coefficient, and low electrical conductivity, such as silica [5,6,7,8,9,10], alumina (Al_2_O_3_) [11,12,13,14], aluminum nitride [15,16,17,18], and hexagonal boron nitride (BN) [19,20,21,22,23,24,25] were studied. Model predictions of and experimental studies on the effects of graphite or graphene oxide on EP resins were also reported [26,27,28].

With the rapid development of the electronics industry, electronic devices are becoming smaller with higher output power, which releases much heat that needs to be dissipated. Therefore, thermal conductivity is one of the most important issues of electronic packaging materials. Normally, the high thermal conductivity of electronic packaging material can be achieved using a high loading of inorganic filler. However, composites with high filler content are usually hard to process. To further increase the thermal conductive properties with satisfactory processing of electronic packaging materials, the combination of different fillers to obtain a perfect thermal conductive network attracted much interest [29,30,31,32,33,34,35,36,37,38]. The use of Al_2_O_3_ particles to reinforce the EP matrix is principally preferred because of its low cost, good thermal stability, corrosive resistance, low thermal expansion, and non-electrical conductivity [11,12,13,14,31,32]. Recently, BN platelets emerged as an outstanding candidate for application to EP-based packaging composite materials because of their unique thermal conductive properties [39]. However, because the boron nitride (BN) filler is anisotropic, with an intrinsic conductivity of 2 W/(m·K) for the through-plane [36] and 400 W/(m·K) for the in-plane [36], the orientation of BN platelets significantly affects the thermal conductivities of the composites. Therefore, many efforts were devoted to control the orientation of BN platelets, including a special preparation process [40], applying the shear force with a doctor blading [41] or a two-roll mill [42], and application of a magnetic field after modifying BN platelets with iron oxides [43,44,45,46]. However, the orientation or alignment of BN platelets in hybrid fillers system is yet to be investigated.

In this paper, the rheological properties and thermal conductivity of EP resin containing two fillers, Al_2_O_3_ particles and BN platelets, were investigated. The combination of the Al_2_O_3_ and the BN into EP resin can have a synergistic effect on the thermal conductivity of the final composite material, because the planar shape of the BN can form the connecting pathway of spherical Al_2_O_3_ particles, thereby improving the thermal conductivity of the composite material. The effect of various amounts of BN on the morphology, rheological properties, and thermal conductivity of the Al_2_O_3_/EP system was carefully studied. It was found that, by adjusting the amount of BN platelets, their orientation can be controlled to facilitate the formation of higher thermally conductive pathways without sacrificing the processability.

## 2. Materials and Methods

### 2.1. Materials

Bisphenol A type epoxy resin, YD-128, was obtained from Kukdo Chemical (Seoul, Korea). Hexahydro-4-methylphthalic anhydride (HMPA), as the hardener for epoxy resin, and 2-ethyl-4-methylimidazole (EMI), as the catalyst, were purchased from Sigma-Aldrich (Yong-In, Korea). Furthermore, 2-(3,4-epoxycyclohexyl)ethyltrimethoxysilane (KBM-303), a coupling agent, was provided by Shln-Etsu Chemical Co., Ltd. (Tokyo, Japan). Aluminum oxides of spherical shapes with a median diameter of 14.4 μm (Al_2_O_3_, DAB-10SI) were purchased from Denki Kagaku Kogyo Kabushiki Kasisha (Tokyo, Japan). Boron nitride filler in the shape of a single-crystal platelet, with a mean particle size of ~45 μm and a surface area of 0.6 m^2^·g^−1^, was purchased from Momentive Performance Materials, Inc. (Waterford, NY, USA). All materials were directly used without purification or modification.

### 2.2. Preparation of the Composite Materials

The composite materials of the epoxy resin filled with alumina and different amounts of BN were prepared by simple mixing with a paste mixer. In a typical procedure, 1.20 g of YD-128, 1.06 g of HMPA, 0.12 g of KBM-303, and 21.60 g of Al_2_O_3_ were mixed together using a paste mixer for 3 min. Subsequently, BN was added to the mixture under continuous mixing for a further 2 min. The cured composites were prepared by adding EMI as a catalyst to the mixture, followed by mixing, molding on a glass mold, and curing at 100 °C for 1 h, with a post cure at 140 °C for 2 h. Table 1 shows the formulated mixtures with different contents of raw material by volume fraction. The numbers in sample codes denote contents of fillers by part per hundred of epoxy resin.

### 2.3. Characterizations of the Composite Materials

The rheological properties of uncured composite materials without catalyst were evaluated using a rheometer, AR2000 (TA Instrument Co., New Castle, DE, USA), equipped with parallel plates having a diameter of 40 mm at 90 °C. In a particular procedure, the dynamic rheological measurement was firstly conducted in an oscillation mode at a constant strain of 5% and a frequency range from 0.1 to 10 Hz. The steady shear measurement was then evaluated with a shear rate range from 0.01 to 10 s^−1^. The stress relaxation was conducted by applying a stress (δ_max_) to attain a strain of 5%; then, the stress was removed, and the relaxation stress versus the time was recorded. Relaxation times were defined on the basis of Maxwell’s model for viscoelastic materials as the time required to relax to 1/e of the initial stress.

The fractured morphology of the composite samples was characterized using a scanning electron microscope (SEM), JEOL JSM-6400 (Akishima, Tokyo, Japan). The composite sample was fractured, and the fractured surface was coated with Pt before observation.

Thermal conductivities of the composite film samples were obtained by multiplying the corresponding thermal diffusivity and volumetric heat capacity. The thermal diffusivity was measured in the direction perpendicular to the flat plane of the film using a Netzsch LFA 447 (Selb Deutschland, Germany) at room temperature. The volumetric heat capacity was calculated by multiplying the material density and specific heat capacity. The density was identified based on the Archimedes principle, and the specific heat capacity of the composite materials was obtained using a differential scanning calorimeter Netzsch DSC 214 (Selb Deutschland, Germany) with a temperature range from 0 to 50 °C and a heating rate of 10 °C/min. 

## 3. Results and Discussion

### 3.1. Characteristics of Fillers

**Figure 1:**
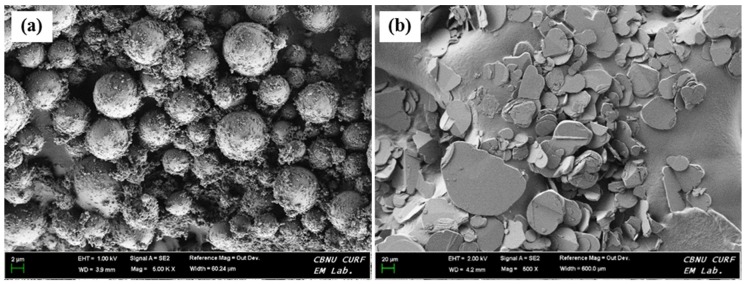
SEM images of alumina (**a**) and crystalline boron nitride (**b**) investigated in this study.

The morphological features of the Al_2_O_3_ spheres and crystalline BN platelets were investigated employing SEM, as shown in Figure 1. A spherical shape and a broad particle-size distribution of the Al_2_O_3_ (from nanometers to several micrometers) were observed (Figure 1a). It is speculated that the broad particle-size distribution of alumina is favorable for the improvement of the flow ability and filler packing in the composite materials. In this context, the small particles can fill in the gaps between the larger particles and increase the maximum packing volume fractions of the particles, thereby reducing the overall viscosity via the Farris effect [47]. Moreover, the Al_2_O_3_ filler is isotropic, with an intrinsic conductivity value of 33 W/(m·K). Figure 1b shows the two-dimensional structure of the BN with side dimensions from several to one hundred micrometers. As mentioned above, due to the anisotropic nature of the BN platelets, their orientation in a polymer matrix significantly affects the thermal conductivity of the composite material.

### 3.2. Rheological Properties of the Composite Materials before Cure

In order to control and optimize process conditions, the rheological study plays an important role. However, rheological behaviors of suspension systems are complex, depending on not only size, shape, and fraction of fillers, but also filler–filler interactions and filler–matrix interactions [48]. 

**Figure 2:**
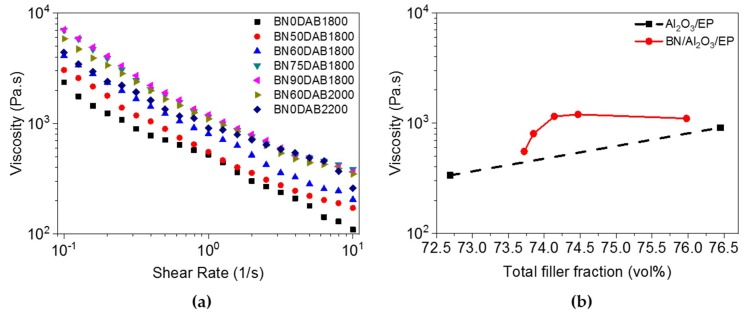
Steady shear viscosities (**a**) and viscosity at a shear rate of 1 s^−1^ vs. total filler fraction of Al_2_O_3_/epoxy resin (EP) and boron nitride (BN)/Al_2_O_3_/EP (**b**).

The flowability of the packaging material is an important factor to evaluate whether it is a suitable candidate to use for packaging electronic devices. As seen in Figure 2a, the viscosities of the composites decrease upon increasing the rate of shear. This is the shear-thinning behavior of non-Newtonian fluids due to the redistribution of fillers and their orientation. The viscosities of the BN/Al_2_O_3_/EP composites strongly depend on the fraction of the BN and Al_2_O_3_. Figure 2b compares the viscosities of the Al_2_O_3_/EP and the BN/Al_2_O_3_/EP composites at the shear rate of 1 s^−1^. The viscosity of the BN/Al_2_O_3_/EP composite increases as the amount of the total filler increases from 73.72 vol % to 74.14 vol %, corresponding to an addition of 3.63 to 5.32 vol % into the Al_2_O_3_/EP mixture. The viscosity of the composite based on the BN/Al_2_O_3_/EP is much higher than that of the composite based on the Al_2_O_3_/EP, because the presence of the BN platelets lowers the flowability of the Al_2_O_3_/EP. However, the orientation of the BN platelets changes from being in a random state to being parallel with the flow of Al_2_O_3_/EP, as shown below in the SEM images, when the total filler content increases from 74.47 to 75.98 vol %, resulting in a slight reduction in viscosity. 

**Figure 3:**
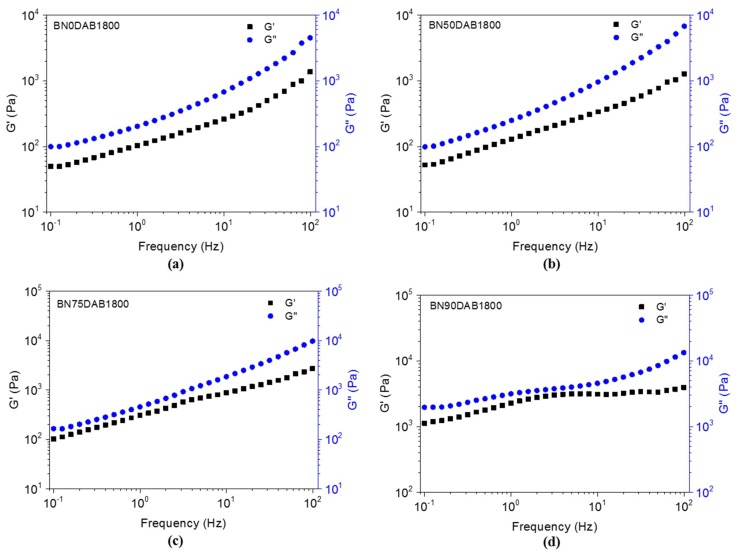
Dynamic modulus versus frequency of (**a**) BN0DAB1800, (**b**) BN50DAB1800, (**c**) BN75DAB1800, and (**d**) BN90DAB1800. See Table 1 for descriptions of composites.

Figure 3 shows typical dynamic modulus curves of the composite based on the Al_2_O_3_/EP and the BN/Al_2_O_3_/EP. The storage modulus represents the elastic deformation, whereas the loss modulus represents the viscous component. It shows that the storage modulus of the composite based on Al_2_O_3_/EP or BN/Al_2_O_3_/EP is lower than loss modulus, showing that the composite behaves like a liquid; that is, the composite materials still flow at an elevated temperature for processing. The modulus of the composites increases upon increasing the amount of BN in the mixture. The slopes of the modulus curves decrease when there is more BN, indicating that the flow ability of the composites decreases and, finally, solid-like behaviors are observed. In particular, when the total filler exceeds 74.47 vol % (BN90DAB1800), the slopes of both G’ and G” sharply decrease, indicating a large reduction in flow ability. This result is consistent with the viscosity measurement.

**Figure 4:**
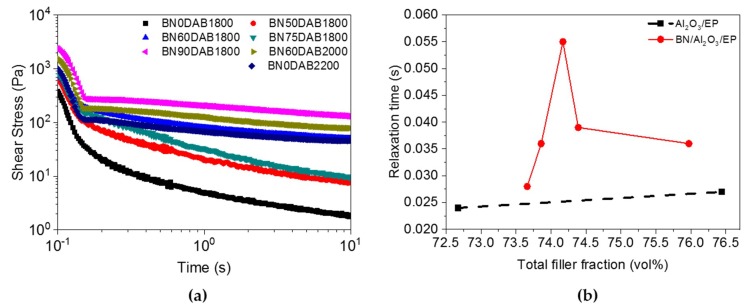
Stress relaxation data (**a**) and relaxation time versus total filler volume fraction of Al_2_O_3_/EP and BN/Al_2_O_3_/EP (**b**).

The viscoelasticity of polymeric materials was investigated by measuring the stress relaxation. A material with a shorter relaxation time is consistent with viscous fluid behavior. The relaxation modulus data of the BN/Al_2_O_3_/EP are shown in Figure 4a. The relaxation time was calculated based on relaxation modulus data, and the relaxation time versus total filler fraction is shown in Figure 4b. It can be seen that the relaxation time also increases with an increased amount of BN; in particular, when the amount of BN increases from 3.63 vol % (BN50DAB1800) to 5.32 vol % (BN75DAB1800), the relaxation time sharply increases. In this surveyed range of BN content, the direction of the BN platelets was random, thereby preventing the flow of the alumina particles and resin mixture. Therefore, the addition of the BN into Al_2_O_3_/EP causes an increase in relaxation time, implying lower flow ability. However, the relaxation time slightly decreases at a high concentration of BN. It seems that the orientation of the BN in the Al_2_O_3_/EP matrix makes the flow easier and reduces the relaxation time. The orientation of the BN platelets at this concentration is confirmed by the SEM images shown below.

### 3.3. Morphology of the Composite Materials

**Figure 5:**
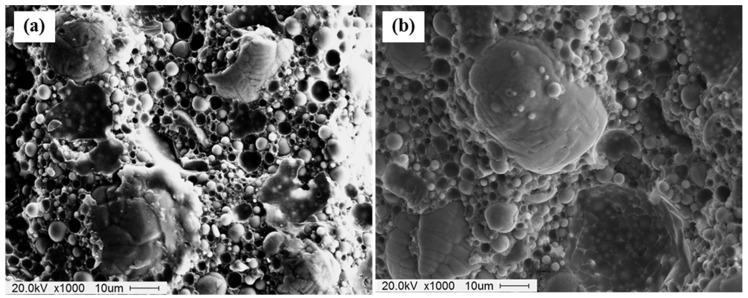
SEM images of Al_2_O_3_/EP without BN: (**a**) BN0DAB1800 and (**b**) BN0DAB2200.

**Figure 6:**
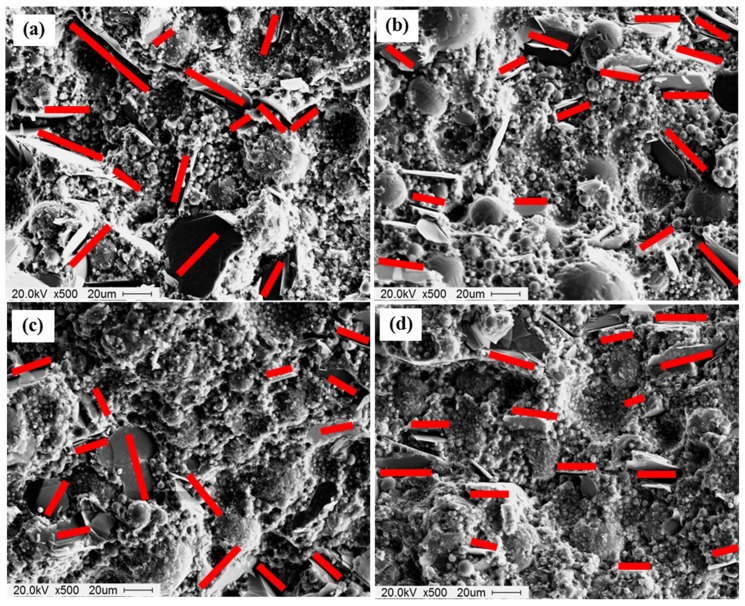
SEM images of Al_2_O_3_/EP filled with (**a**) 3.63 vol %, (**b**) 4.30 vol %, (**c**) 5.32 vol %, and (**d**) 6.32 vol % boron nitride.

The SEM images of the Al_2_O_3_/EP composite without and with the addition of the BN filler are shown in Figure 5 and Figure 6, respectively. It is clearly seen that the Al_2_O_3_ spheres are well dispersed within the polymer matrix (Figure 5). Interestingly, the larger Al_2_O_3_ spheres are surrounded by the smaller ones, because the broad size distribution of the Al_2_O_3_ spheres minimizes the distance between particles. Meanwhile, the thermal conductivity of the composite mainly results from the EP resin contribution with low thermal conductivity; the closer distances between the filler particles can reduce the thickness of the EP resin layer, leading to easily conductive pathways for the composite samples.

**Scheme 1:**
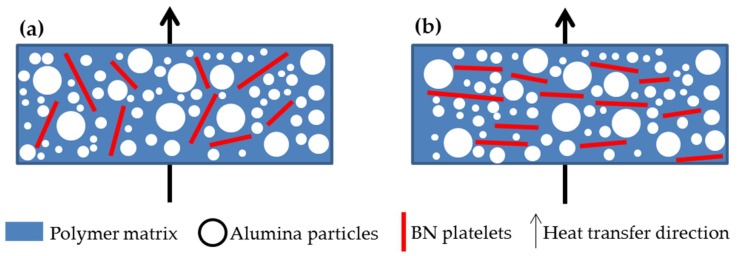
Orientation of the BN platelets in the polymer matrix at (**a**) low content and (**b**) high content of BN.

The addition of fillers with different shapes into an EP matrix can increase the packing of the obtained composites by making it easier to form various conductive pathways. Therefore, the morphology of the Al_2_O_3_/EP composite sample with the addition of BN was investigated, as shown in Figure 6. The distribution and orientation of the BN are shown as red bars. At a low concentration of BN, the BN platelets are randomly dispersed into the Al_2_O_3_/EP matrix without orientation (Figure 6a–c). Therefore, thermal conductivity pathways of BN platelets are in-plane (Scheme 1a). However, the BN platelets prefer a parallel arrangement with the surface of the composite film to facilitate their movement between the spherical Al_2_O_3_ particles as the BN amount increases (Figure 6d); hence, thermal conductivity pathways are through-plane (Scheme 1b). The change in the orientation of BN platelets results in a reduction in the thermal conductivity of the composite films, as well as in the viscosity of the composites.

### 3.4. Thermal Conductivity of the Composite Materials

**Figure 7:**
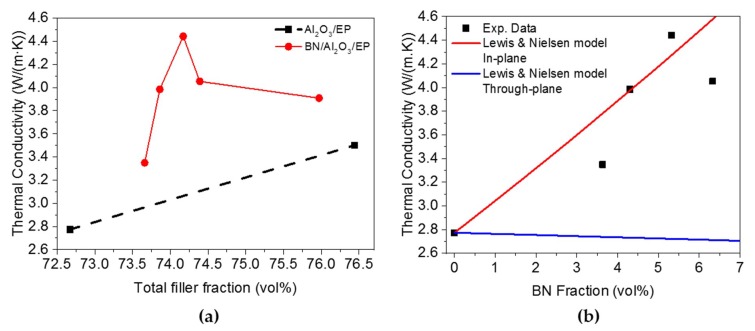
Thermal conductivity of the composite materials versus total filler fraction (**a**), and comparison of experimental data and the model predictions as a function of BN fraction (**b**).

The thermal conductivity of the composites strongly depends on the filler concentration, because of the high conductivity of the filler. However, the viscosity of the composite increases as the amount of filler increases; thus, the materials cannot be processed. The simultaneous use of two types of fillers, including Al_2_O_3_ and BN, in the EP matrix is believed to further improve the thermal conductivity of the composite materials. Figure 7a clearly shows the strong effect of BN content on the thermal conductivity of the composite. The thermal conductivity of the BN/Al_2_O_3_/EP composite is higher than that of the Al_2_O_3_/EP composite. The thermal conductivity increases from 2.77 W/(m·K) for the composite without BN (BN0DAB1800) to 3.35 W/(m·K) for the composite with 3.63 vol % (BN50DAB1800) BN loading. When the BN amount increases from 3.63 (BN50DAB1800) to 5.32 vol % (BN75DAB1800), the thermal conductivity increases sharply, from 3.35 W/(m·K) to 4.44 W/(m·K), that is, 1.6 times higher than that of the composite film without BN. However, the thermal conductivity is then reduced to 4.05 W/m·K when the BN concentration increases to 6.32 vol % (BN90DAB1800), probably because the high BN content in the BN90DAB1800 composite samples alters the BN orientation from being random to being parallel, thereby changing of the thermal conductive pathways of BN platelets from in-plane into through-plane (Scheme 1). In this case, the thermal conduction pathways of the BN platelets mainly happen through-plane, which has a low intrinsic conductivity, thus reducing the overall conductivity of the composite. The orientation of the BN platelets is clearly shown in the SEM images (Figure 6). 

Lewis and Nielson modified the Halpin–Tsai equation for transport properties, such as the thermal conductivity of polymer composites [49].

(1) $$k_{c}=k_{m}\left(\frac{1+AB\emptyset }{1-B\emptyset \psi }\right),$$

(2) $$B=\frac{\frac{k_{f}}{k_{m}}-1}{\frac{k_{f}}{k_{m}}+A},$$

(3) $$\psi =1+\left(\frac{1-\phi_{m}}{\phi_{m}^{2}}\right)\phi ,$$

where $k_{c}$, $k_{m}$, and $k_{f}$ represent the thermal conductivity of the composite, polymer matrix, and filler, respectively, and ∅ is the volume fraction of filler. The value of *A* in Equation (1) is a function of the orientation and shape of the filler, and $\emptyset_{m}$ is the maximum packing fraction of the filler. According to Corcione and Maffezzoli, the prediction by Equation (1) showed reasonably good agreement with the experimental data of composites filled with platelet fillers such as graphene [27]. In order to study the model predictions on the effects of BN in the hybrid composites, the thermal conductivity of Al_2_O_3_/EP (BN0DAB1800) was used as the value of $k_{m}$ in Equation (1) and parameters of BN were employed to investigate the prediction by the model on the effects of BN. Due to the anisotropic nature of BN platelets, $k_{f}$ = 400 W/(m·K) and *A* = 10 were used for the model prediction of the in-plane thermal conduction, while $k_{f}$ = 2 (W/(m·K) and *A* = 0.5 were used for the model prediction of the through-plane conduction by BNs [49]. The maximum packing fraction ($\phi_{m}$) of thin platelets was assumed as 0.85 [50]. Based on these considerations, the experimentally determined thermal conductivity data and the predictions by the model for the in-plane thermal conduction and that for the through-plane conduction by BN are given in Figure 7b. It is observed that, as the BN content increases from 3.63 to 5.32 vol %, the experimentally determined thermal conductivities of the composites closely follow the model prediction based on the in-plane thermal conduction (red line). However, with increasing BN content further, the experimental data begin deviating from the prediction based on the in-plane thermal conduction due to the change of the thermal conduction pathway by BN from in-plane to through-plane, as shown in Scheme 1. 

## 4. Conclusions

This study investigated the morphology and properties of an Al_2_O_3_/EP composite film filled with different amounts of BN filler. The viscosity of the obtained composites slightly increased with incorporation of the BN filler. The obtained flow properties of the composites were demonstrated to be suitable for applicable processing. The thermal conductivity of the BN/Al_2_O_3_/EP composites was significantly improved by adding a small amount of the BN up to 5.32 vol % without a significant effect on the flow ability for processing. The use of a higher BN amount reduced the thermal conductivity of the composite because of the alteration of the thermal conductive pathway of BN platelets from in-plane to through-plane. The orientation of BN platelets can be controlled by adjusting their loading to facilitate the formation of higher thermally conductive pathways.

## Figures and Tables

**Table 1:** Sample codes and formulation of epoxy resins filled with alumina and boron nitride (BN) (by volume). YD-128—bisphenol A epoxy resin; HMPA—hexahydro-4-methylphthalic anhydride; KBM-303—2-(3,4-epoxycyclohexyl)ethyltrimethoxysilane; EMI—2-ethyl-4-methylimidazole.

Sample Codes	YD-128	HMPA	KBM-303	EMI	Alumina	BN	Total Filler
BN0DAB1800	13.34	12.18	1.50	0.29	72.69	0	72.69
BN50DAB1800	12.86	11.71	1.43	0.28	70.09	3.63	73.72
BN60DAB1800	12.76	11.67	1.44	0.28	69.55	4.30	73.85
BN75DAB1800	12.61	11.52	1.45	0.28	68.82	5.32	74.14
BN90DAB1800	12.50	11.37	1.40	0.27	68.14	6.33	74.47
BN60DAB2000	11.89	10.82	1.32	0.25	71.96	4.02	75.98
BN0DAB2200	11.48	10.52	1.30	0.25	76.45	0	76.45

## References

[1] Ho T.H., Wang C.S. (2001). Modification of epoxy resin with siloxane containing phenol aralkyl epoxy resin for electronic encapsulation application. Eur. Polym. J..

[2] Rimdusit S., Ishida H. (2000). Development of new class of electronic packaging materials based on ternary systems of benzoxazine, epoxy, and phenolic resins. Polymer.

[3] Tai H.J., Wang J.B., Chen J.H., Chou H.L. (2001). Synthesis and properties of vinyl siloxane modified cresol novolac epoxy for electronic encapsulation. J. Appl. Polym. Sci..

[4] Wang C.S., Mendoza A. (1991). 2,6-dibromo-3,5-dimethyl-4-hydroxybenzyl ether and epoxy systems—Their application in electronic packaging. Polym. Bull..

[5] Dueramae I., Jubsilp C., Takeichi T., Rimdusit S. (2014). High thermal and mechanical properties enhancement obtained in highly filled polybenzoxazine nanocomposites with fumed silica. Compos. Part B Eng..

[6] Heo G.Y., Park S.J. (2012). Effect of coupling agents on thermal, flow, and adhesion properties of epoxy/silica compounds for capillary underfill applications. Powder Technol..

[7] Ng F.C., Abas A., Gan Z.L., Abdullah M.Z., Ani F.C., Ali M.Y.T. (2017). Discrete phase method study of ball grid array underfill process using nano-silica filler-reinforced composite-encapsulant with varying filler loadings. Microelectron. Reliab..

[8] Weltevreden E.R., Tesarski S.J., Wymyslowski A., Erinc M., Gielen A.W.J. (2012). A multi-scale approach of the thermo-mechanical properties of silica-filled epoxies used in electronic packaging. Microelectron. Reliab..

[9] Sun Y.Y., Zhang Z.Q., Wong C.P. (2006). Study and characterization on the nanocomposite underfill for flip chip applications. IEEE Trans. Compon. Packag. Technol..

[10] Teh P.L., Mariatti M., Wagiman A.N.R., Beh K.S. (2008). Effect of curing agent on the properties of mineral silica filled epoxy composites. Polym. Compos..

[11] Agrawal A., Satapathy A. (2015). Effect of Al_2_O_3_ addition on thermo-electrical properties of polymer composites: An experimental investigation. Polym. Compos..

[12] Jeong U.S., Lee Y.J., Shin D.G., Lim H.M., Mun S.Y., Kwon W.T., Kim S.R., Kim Y.H., Shim K.B. (2015). Highly thermal conductive alumina plate/epoxy composite for electronic packaging. Trans. Electr. Electron. Mater..

[13] Kim D.J., Kang P.H., Nho Y.C. (2004). Characterization of mechanical properties of gamma Al_2_O_3_ dispersed epoxy resin cured by gamma-ray radiation. J. Appl. Polym. Sci..

[14] McGrath L.M., Parnas R.S., King S.H., Schroeder J.L., Fischer D.A., Lenhart J.L. (2008). Investigation of the thermal, mechanical, and fracture properties of alumina-epoxy composites. Polymer.

[15] Lee E.S., Lee S.M., Shanefield D.J., Cannon W.R. (2008). Enhanced thermal conductivity of polymer matrix composite via high solids loading of aluminum nitride in epoxy resin. J. Am. Ceram. Soc..

[16] Yu S.Z., Hing P., Hu X. (2002). Thermal conductivity of polystyrene-aluminum nitride composite. Compos. Part A Appl. Sci. Manuf..

[17] Xu Y.S., Chung D.D.L., Mroz C. (2001). Thermally conducting aluminum nitride polymer-matrix composites. Compos. Part A Appl. Sci. Manuf..

[18] Zhou Y.C., Wang H., Wang L., Yu K., Lin Z.D., He L., Bai Y.Y. (2012). Fabrication and characterization of aluminum nitride polymer matrix composites with high thermal conductivity and low dielectric constant for electronic packaging. Mater. Sci. Eng. B.

[19] Isarn I., Massagues L., Ramis X., Serra A., Ferrando F. (2017). New BN-epoxy composites obtained by thermal latent cationic curing with enhanced thermal conductivity. Compos. Part A Appl. Sci. Manuf..

[20] Donnay M., Tzavalas S., Logakis E. (2015). Boron nitride filled epoxy with improved thermal conductivity and dielectric breakdown strength. Compos. Sci. Technol..

[21] Du B.X., Xiao M., Zhang J.W. Effect of thermal conductivity on tracking failure of epoxy/BN composite under pulse strength. Proceedings of the IEEE International Conference on Solid Dielectrics.

[22] Wang Z.B., Iizuka T., Kozako M., Ohki Y., Tanaka T. (2011). Development of epoxy/BN composites with high thermal conductivity and sufficient dielectric breakdown strength part I -Sample Preparations and Thermal Conductivity. IEEE Trans. Dielectr. Electr. Insul..

[23] Xiao M., Du B.X. (2016). Effects of high thermal conductivity on temperature rise of epoxy cast windings for power transformer. IEEE Trans. Dielectr. Electr. Insul..

[24] Lin Z.Y., Mcnamara A., Liu Y., Moon K.S., Wong C.P. (2014). Exfoliated hexagonal boron nitride-based polymer nanocomposite with enhanced thermal conductivity for electronic encapsulation. Compos. Sci. Technol..

[25] Wattanakul K., Manuspiya H., Yanumet N. (2011). Effective surface treatments for enhancing the thermal conductivity of bn-filled epoxy composite. J. Appl. Polym. Sci..

[26] Acocella M.R., Corcione C.E., Giuri A., Maggio M., Guerra G., Maffezzoli A. (2017). Catalytic activity of oxidized carbon black and graphene oxide for the crosslinking of epoxy resins. Polymers.

[27] Corcione C.E., Maffezzoli A. (2013). Transport properties of graphite/epoxy composites: Thermal, permeability and dielectric characterization. Polym. Test..

[28] Acocella M.R., Corcione C.E., Giuri A., Maggio M., Maffezzoli A., Guerra G. (2016). Graphene oxide as a catalyst for ring opening reactions in amine crosslinking of epoxy resins. RSC Adv..

[29] Chen C., Tang Y.J., Ye Y.S., Xue Z.G., Xue Y., Xie X.L., Mai Y.W. (2014). High-performance epoxy/silica coated silver nanowire composites as underfill material for electronic packaging. Compos. Sci. Technol..

[30] Zha J.W., Zhu T.X., Wu Y.H., Wang S.J., Li R.K.Y., Dang Z.M. (2015). Tuning of thermal and dielectric properties for epoxy composites filled with electrospun alumina fibers and graphene nanoplatelets through hybridization. J. Mater. Chem. C.

[31] Choi S., Kim J. (2013). Thermal conductivity of epoxy composites with a binary-particle system of aluminum oxide and aluminum nitride fillers. Compos. Part B Eng..

[32] Sanada K., Tada Y., Shindo Y. (2009). Thermal conductivity of polymer composites with close-packed structure of nano and micro fillers. Compos. Part A Appl. Sci. Manuf..

[33] Wetzel B., Haupert F., Zhang M.Q. (2003). Epoxy nanocomposites with high mechanical and tribological performance. Compos. Sci. Technol..

[34] Hong J.P., Yoon S.W., Hwang T., Oh J.S., Hong S.C., Lee Y., Nam J.D. (2012). High thermal conductivity epoxy composites with bimodal distribution of aluminum nitride and boron nitride fillers. Thermochim. Acta.

[35] Kumari L., Zhang T., Du G.H., Li W.Z., Wang Q.W., Datye A., Wu K.H. (2008). Thermal properties of CNT-Alumina nanocomposites. Compos. Sci. Technol..

[36] Lee G.W., Park M., Kim J., Lee J.I., Yoon H.G. (2006). Enhanced thermal conductivity of polymer composites filled with hybrid filler. Compos. Part A Appl. Sci. Manuf..

[37] Yu W., Xie H.Q., Yin L.Q., Zhao J.C., Xia L.G., Chen L.F. (2015). Exceptionally high thermal conductivity of thermal grease: Synergistic effects of graphene and alumina. Int. J. Therm. Sci..

[38] Su J.L., Xiao Y., Ren M. (2013). Enhanced thermal conductivity in epoxy nanocomposites with hybrid boron nitride nanotubes and nanosheets. Phys. Status Solidi A.

[39] Sichel E.K., Miller R.E., Abrahams M.S., Buiocchi C.J. (1976). Heat-capacity and thermal-conductivity of hexagonal pyrolytic boron-nitride. Phys. Rev. B.

[40] Yu C.P., Zhang J., Li Z., Tian W., Wang L.J., Luo J., Li Q.L., Fan X.D., Yao Y.G. (2017). Enhanced through-plane thermal conductivity of boron nitride/epoxy composites. Compos. Part A Appl. Sci. Manuf..

[41] Shen H., Guo J., Wang H., Zhao N., Xu J. (2015). Bioinspired modification of h-BN for high thermal conductive composite films with aligned structure. ACS Appl. Mater. Interfaces.

[42] Kuang Z.Q., Chen Y.L., Lu Y.L., Liu L., Hu S., Wen S.P., Mao Y.Y., Zhang L.Q. (2015). Fabrication of highly oriented hexagonal boron nitride nanosheet/elastomer nanocomposites with high thermal conductivity. Small.

[43] Cho H.B., Tokoi Y., Tanaka S., Suematsu H., Suzuki T., Jiang W.H., Niihara K., Nakayama T. (2011). Modification of BN nanosheets and their thermal conducting properties in nanocomposite film with polysiloxane according to the orientation of BN. Compos. Sci. Technol..

[44] Lim H.S., Oh J.W., Kim S.Y., Yoo M.J., Park S.D., Lee W.S. (2013). Anisotropically alignable magnetic boron nitride platelets decorated with iron oxide nanoparticles. Chem. Mater..

[45] Yuan C., Duan B., Li L., Xie B., Huang M.Y., Luo X.B. (2015). Thermal conductivity of polymer-based composites with magnetic aligned hexagonal boron nitride platelets. ACS Appl. Mater. Interfaces.

[46] Lin Z.Y., Liu Y., Raghavan S., Moon K.S., Sitaraman S.K., Wong C.P. (2013). Magnetic alignment of hexagonal boron nitride platelets in polymer matrix: Toward high performance anisotropic polymer composites for electronic encapsulation. ACS Appl. Mater. Interfaces.

[47] Farris R.J. (1968). Prediction of the viscosity of multimodal suspensions from unimodal viscosity data. Trans. Soc. Rheol..

[48] Han C.D. (2007). Rheology and Processing of Polymeric Materials.

[49] Kumlutas D., Tavman I.H., Coban M.T. (2003). Thermal conductivity of particle filled polyethylene composite materials. Compos. Sci. Technol..

[50] Hill R.F., Supancic P.H. (2002). Thermal conductivity of platelet-filled polymer composites. J. Am. Ceram. Soc..

